# Supplementary material for: Preliminary evidence from a multicenter prospective observational study of the safety and efficacy of chloroquine for the treatment of COVID-19
Source: Natl Sci Rev. 2020 May 28;7(9):1428–36. doi: 10.1093/nsr/nwaa113 (PMC7313782; doi:10.1093/nsr/nwaa113)
Supplement: nwaa113_Supplemental_File [file nwaa113_supplemental_file.docx]

**Supplemental Materials**

**Supplementary Tables**

**Supplementary Table 1.** Detailed information for the nine patients who decided to exit the study before treatment completion.

**Supplementary Table 2.** Summary of outcomes in patients with moderate COVID-19 symptoms.

**Supplementary Table 3.** Summary of outcomes in patients from the Fifth Affiliated Hospital of Sun Yat-sen University in Zhuhai, Guangdong, China. **Supplementary Table 4.** Summary of outcomes in patients receiving half dose of chloroquine or in non-chloroquine group from Guangdong Province.

**Supplementary Table 5.** Summary of adverse events in patients who are older than 65 or with pre-existing conditions

**Supplementary Table 6.** Summary of patients receiving non-chloroquine treatment for COVID-19 in the historical control group.

**Supplementary Table 7.** History of changes to the protocol.

**Supplementary Table 8.** Number of patients by treatment group and by center.

**Supplementary Figures**

**Supplementary Figure 1.** Kaplan-Meier plot of the duration of fever in the overall population with confirmed SARS-CoV-2 infection.

**Supplementary Figure 2.** Kaplan-Meier plot of the length of hospital stay in the overall population with confirmed SARS-CoV-2 infection.

**Supplementary Figure 3.** The distribution of log-transformed serum concentration level in patients treated with chloroquine from SYSU5 before and after treatment completion (N=50).

**Supplementary Figure 4.** The Kaplan-Meier plot on the time to undetectable viral RNA stratified by tertiles of log-transformed maximum observed value of serum concentration of chloroquine (N=50).

**Supplementary Methods**

Definition of outcomes

Measurement of serum concentration of Chloroquine

Supplementary Table 9

Supplementary Table 10

Supplementary Table 11

Quantitative viral RNA testing

Original protocol, study flow, adverse events reporting guidelines

**Supplementary Table**

**Supplemental Table 1. Detailed information for the nine patients who decided to exit the study before treatment completion.**

| **Index** | **Sex** | **Age** | **Clinial symptoms** | **Reasons for withdrawing** |
| --- | --- | --- | --- | --- |
| 1 | M | 56 | moderate | Intolerance of nausea and vomiting |
| 2 | F | 36 | moderate | Intolerance of dizziness; combination of Abidol |
| 3 | F | 32 | moderate | Intolerance of palpitation discomfort |
| 4 | M | 50 | moderate | Mild hemolysis |
| 5 | M | 26 | mild | ECG suggesting prolonged QT interval |
| 6 | F | 45 | moderate | Intolerance of tinnitus |
| 7 | M | 51 | moderate | Intolerance of gastrointestinal discomfort |
| 8 | F | 62 | moderate | Intolerance of gastrointestinal discomfort |
| 9 | F | 59 | mild | Depression as preexisting condition |

**Supplementary Table 2. Summary of outcomes in patients with moderate COVID-19 symptoms**^§^**.**

|  | **chloroquine**  **(N=184)** | **Non-chloroquine**  **(N=157)** | **Difference**  **(95% CI)** ^†^ |
| --- | --- | --- | --- |
| Interval time from symptom onset to treatment initiation, median (IQR) | 12.0 (6.0, 19.0) | 7.0 (3.0, 13.0) | 5.0 (3.0, 7.5) |
| Time to undetectable viral RNA, median no. of days (IQR) | 3.0 (3.0, 5.0) | 8.0 (6.0, 12.0) | -5.0 (-6.0, -4.0) |
| Patients with undetectable viral RNA by, N (%) |  | | |
| Day 10 | 168.0 (91.3) | 92.0 (58.6) | 32.7 (23.9, 42.1) |
| Day 14 | 176.0 (95.7) | 126.0 (80.3) | 15.4 (7.8, 22.6) |
| Duration of fever, no. of days, geometric mean (CV) * | 1.2 (48.3) | 1.6 (96.0) | 0.8 (0.5, 0.9) |
| Length of hospital stay, median no. of days (IQR) | 19.0 (16.0, 23.0) | 20.0 (15.0, 23.0) | -1.0 (-2.5, 0.0) |
| Patients aggravate to severe symptoms during study period, N (%) | 1.0 (0.6) | 6.0 (3.8) | -3.2 (-7.0, 1.4) |
| Patients with adverse events during study period, N (%) | 50.0 (27.2) | 49.0 (31.2) | -4.0 (-14.0, 6.0) |

Abbreviations: CI, confidence interval; IQR, inter-quartile range; CV, coefficient of variation.

^§^ Definitions of outcomes are listed in Supplemental Methods.

^†^ 95% CI for continuous variables are calculated by bootstrapping. 95% CI for binary variables are calculated with Wilson method. The difference for duration of fever is geometric mean ratio of chloroquine group to non-chloroquine group. The differences for all other variables are the absolute difference between chloroquine group and non-chloroquine group.

* The number of patients had at least one day of fever is 37 and 39 in the chloroquine and non-chloroquine group respectively.

**Supplementary Table 3. Summary of outcomes in patients from the Fifth Affiliated Hospital of Sun Yat-sen University in Zhuhai, Guangdong Province**^§^**.**

|  | **chloroquine**  **(N=50)** | **Non-chloroquine**  **(N=21)** | **Difference (95% CI)** ^†^ |
| --- | --- | --- | --- |
| Interval time from symptom onset to treatment initiation, median (IQR) | 7.0 (2.0, 10.0) | 6.0 (4.0, 8.0) | 1.0 (-3.0, 4.0) |
| Time to undetectable viral RNA, median no. of days (IQR) | 4.5 (2.0, 8.0) | 8.0 (4.0, 11.0) | -3.5 (-6.0, 1.0) |
| Patients with undetectable viral RNA by, N (%) |  | | |
| Day 10 | 45.0 (90.0) | 15.0 (71.4) | 18.6 (-6.0, 39.2) |
| Day 14 | 50.0 (100.0) | 18.0 (85.7) | 14.3 (-8.8, 28.1) |
| Patients with improved chest CT by, N (%)^‡^ |  | | |
| Day 10 | 18.0 (48.6) | 7.0 (38.9) | 9.7 (-16.0, 35.6) |
| Day 14 | 27.0 (73.0) | 12.0 (66.7) | 6.3 (-22.2, 32.0) |
| Duration of fever, no. of days, geometric mean (CV) * | 1.2 (49.6) | 2.6 (121.2) | 0.5 (0.2, 0.8) |
| Hospital stay, median no. of days (IQR) | 20.0 (15.0, 29.0) | 17.0 (15.0, 21.0) | 3.0 (-1.0, 7.0) |
| Duration of oxygen support, median no. of days (IQR) | 7.5 (2.0, 12.3) | 6.5 (5.0, 12.3) | 0.4 (-5.0, 4.5) |
| Patients with adverse events during study period, N (%) | 20.0 (40.0) | 9.0 (42.9) | -2.9 (-28.0, 23.4) |
| Re-positive patients after discharge by day 7 | 3.0 (6.0) | 0.0 (0.0) | 6.0 (-5.6, 25.8) |

Abbreviations: CI, confidence interval; IQR, inter-quartile range; CV, coefficient of variation.

^§^ Definitions of outcomes are listed in Supplemental Methods.

^†^ 95% CI for continuous variables are calculated by bootstrapping. 95% CI for binary variables are calculated with Wilson method. The difference for duration of fever is geometric mean ratio of chloroquine group to non-chloroquine group. The differences for all other variables are the absolute difference between chloroquine group and non-chloroquine group.

* The number of patients had at least one day of fever is 16 and 10 in the chloroquine and non-chloroquine group respectively.

^‡^The total number of patients had abnormal chest CT image at baseline is 37 and 18 in the chloroquine and non-chloroquine group respectively.

**Supplemental Table 4. Summary of outcomes in patients receiving half dose of chloroquine or in non-chloroquine group from Guangdong Province.**

|  | **chloroquine**  **(N=29)** | **Non-chloroquine**  **(N=96)** | **Difference (95% CI)** |
| --- | --- | --- | --- |
| Interval time from symptom onset to treatment initiation, median (IQR) | 7.0 (5.0, 13.0) | 4.0 (2.0, 7.0) | 3.0 (1.0, 7.0) |
| Time to undetectable viral RNA, median no. of days (IQR) | 5.0 (2.0, 8.0) | 9.0 (5.8, 14.0) | -4.0 (-8.0, -2.0) |
| Patients with undetectable viral RNA by, N (%) |  |  |  |
| Day 10 | 25.0 (86.2) | 50.0 (52.1) | 34.1 (20.2, 55.5) |
| Day 14 | 26.0 (89.7) | 71.0 (74.0) | 15.7 (3.0, 35.6) |
| Duration of fever, no. of days, geometric mean (CV) | 1.0 (0.0) | 2.3 (116.3) | 0.4 (0.3, 0.6) |
| Length of hospital stay, median no. of days (IQR) | 19.0 (15.0, 24.0) | 18.0 (13.8, 25.0) | 1.5 (-2.0, 7.0) |
| Patients with adverse events during study period, N (%) | 1.0 (3.5) | 16.0 (16.7) | -13.2 (-30.7, -3.4) |

Abbreviations: CI, confidence interval; IQR, inter-quartile range; CV, coefficient of variation.

^§^ Definitions of outcomes are listed in Supplemental Methods.

^†^ 95% CI for continuous variables are calculated by bootstrapping. 95% CI for binary variables are calculated with Wilson method. The difference for duration of fever is geometric mean ratio of chloroquine group to non-chloroquine group. The differences for all other variables are the absolute difference between chloroquine group and non-chloroquine group.

* The number of patients had at least one day of fever is 16 and 10 in the chloroquine and non-chloroquine group respectively.

**Supplementary Table 5. Summary of adverse events in patients who are older than 65 or with pre-existing conditions**

| **Conditions** | **True, N (%)** | **False, N (%)** | **Difference (95% CI)** |
| --- | --- | --- | --- |
| Age > 65 | 3.0 (43.0) | 50.0 (26.0) | 17.0 (-28.0, 61.0) |
| Hypertension | 6.0 (46.0) | 31.0 (48.0) | -2.0 (-32.0, 27.0) |
| Type 2 diabetes | 2.0 (50.0) | 35.0 (47.0) | 3.0 (-40.0, 45.0) |

**Supplementary Table 6. Summary of patients receiving non-chloroquine treatment for COVID-19 in the historical control group.**

| **Non-chloroquine** | **Numbers** | **%** |
| --- | --- | --- |
| Ab | 83 | 47 |
| LPv/r+INF | 5 | 3 |
| LPv/r+INF+Ab | 18 | 10 |
| LPv/r+INF+Ab+CTM | 6 | 3 |
| LPv/r+INF+Ab+CTM+Os | 3 | 2 |
| LPv/r+INF+CTM | 1 | 1 |
| LPv/r+INF+CTM+Os | 3 | 2 |
| LPv/r | 28 | 16 |
| LPv/r+Ab | 23 | 13 |
| LPv/r+CTM | 1 | 1 |
| Reg | 5 | 3 |

Abbreviations: Ab, arbidol hydrochloride; LPv/r, Lopinavir Ritonavir; INF, interferon; CTM, Chinese Traditional Medicine; Reg, regular (No antiviral drugs)

For patients in the historical control group, clinicians chose the therapy according to the combination of patient’s will and condition following the most updated version of China’s Novel Coronavirus Pneumonia Diagnosis and Treatment Plan. The treatment choices include either no anti-viral drugs or one or more of the following anti-viral drugs, three times-daily oral of 500mg Arbidol (Suzhou pharmaceutical factory of Jiangsu Wuzhong Pharmaceutical Group Co., Ltd), twice to three times-daily intravenous infusions of 500mg ribavirin, twice-daily aerosol inhalation of 5 million units alpha-interferon or twice-daily oral of 400/100mg lopinavir/ritonavir (AbbVie Deutschland GmbH & Co. KG).

**Supplementary Table 7. History of changes to the protocol.**

| **Version** | **Date** | **Section** | **Section title** | **Previous Vision** | **Revision** | **Reason** |
| --- | --- | --- | --- | --- | --- | --- |
| V1→V2 | 2020-2-6 | 3.4 | Methods | None | we monitor the serum concentration of chloroquine at day 1, 3, 5, 7, 10 during drug administration and day 1 to 7, and day 14, day 21 after treatment completion | To evaluate the serum concentration of chloroquine phosphate and drug accumulation and toxicity |
|  |  | 4.1 | Tested drug | Chloroquine phosphate tablets 0.5 g twice a day | Chloroquine phosphate tablets 0.5 g once/twice a day, for 10 days | Specify the usage and dosage of chloroquine phosphate |
|  |  | 4.1 | Tested drug | Prohibition of combination with chloroquine phosphate : digitalis, amiodarone, domperidone, droperidol, haloperidol, clarithromycin, methadone, procainamide, hydrochlorothiazide, sparfloxacin, cisapride, indapamide, | Prohibition of combining chloroquine phosphate with following drugs: digitalis, amiodarone, domperidone, droperidol, haloperidol, clarithromycin, methadone, procainamide, hydrochlorothiazide, sparfloxacin, levofloxacin, moxifloxacin, cisapride, indapamide. | 1. Add prohibition of combining chloroquine phosphate with moxifloxacin.  2. The adverse reactions of moxifloxacin combined with chloroquine phosphate in the digestive tract were significantly increased |
|  |  | 4.1 | Tested drug | Lopinavir / ritonavir tablets | Removal of the Lopinavir / ritonavir treatment group | Number of new COVID-19 patients decreased |
| V2→V3 | 2020-2-8 | 4.2 | Duration of follow-up | 28 days | 30 days | Prolong the observation period in order to clarify the adverse reactions of chloroquine phosphate |
|  |  | 2.1 | Secondary outcome | 1. To explore the safety of chloroquine phosphate in the treatment of COVID-19.  2. Establish a standardized guideline for treating COVID-19 with chloroquine phosphate | 1. To explore the safety of chloroquine phosphate in the treatment of COVID-19.  2. Establish a standardized guideline for treating COVID-19 with chloroquine phosphate  3. To explore the correlation between the steady-state serum concentration of chloroquine phosphate and the therapeutic effect and adverse reactions. | In order to fully evaluate the safety of chloroquine phosphate, we tested serum concentration of chloroquine phosphate. |
|  |  | 4.5 | Assessment adverse events of tested drugs | Assess possible associations between adverse events and chloroquine phosphate or lopinavir/ritonavir | Assessment of possible association between adverse events and serum concentration of chloroquine phosphate | We did not include lopinavir/ritonavir as subjects |

**Supplementary Table 8. Number of patients by treatment group and by center.**

|  | **case** | **control** |
| --- | --- | --- |
| Dongguan Ninth People's Hospital | 15 | 13 |
| Wuhan East West Lake Mobile Cabin Hospitals | 79 | 80 |
| The Fifth Affiliated Hospital, Sun Yat-sen University | 50 | 21 |
| Zhongshan Second People's Hospital | 6 | 0 |
| Foshan first people's Hospital | 1 | 12 |
| Foshan Fourth People's Hospital | 0 | 12 |
| Guangdong Second People's Hospital | 0 | 6 |
| Guangzhou Eighth People's Hospital | 29 | 11 |
| Huizhou Central People's Hospital, | 4 | 0 |
| Shenzhen Third People's Hospital | 11 | 0 |
| Qingyuan people's Hospital | 1 | 8 |
| Maoming people's Hospital, | 1 | 13 |

**Supplementary Figures**

**Supplementary Figure 1. Kaplan-Meier plot of the duration of fever in the overall population with confirmed SARS-CoV-2 infection.**


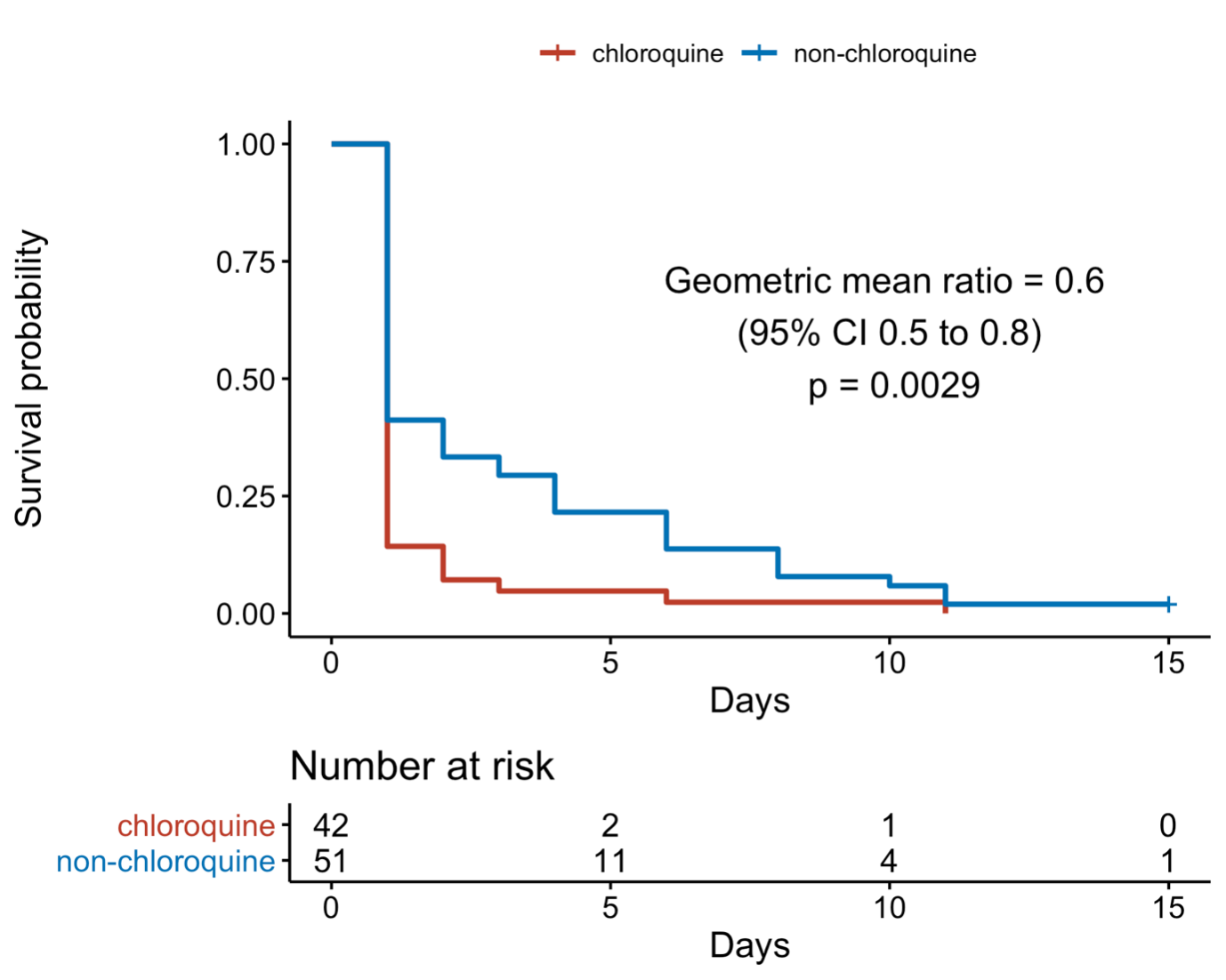


**Supplementary Figure 2. Kaplan-Meier plot of the length of hospital stay in the overall population with confirmed SARS-CoV-2 infection.**


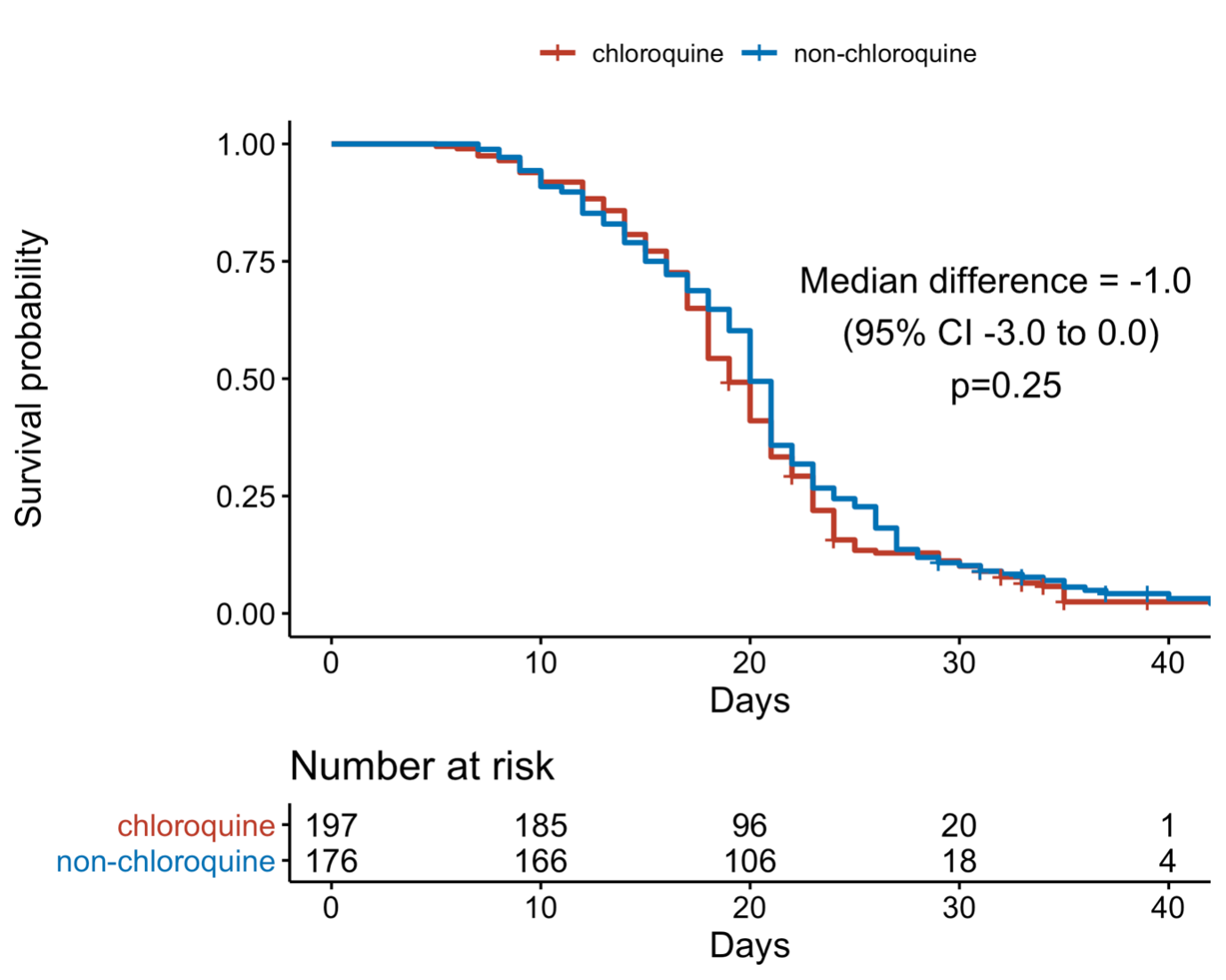


**Supplementary Figure 3. The distribution of log-transformed serum concentration level in patients treated with chloroquine from SYSU5 before and after treatment completion (N=50).**

**
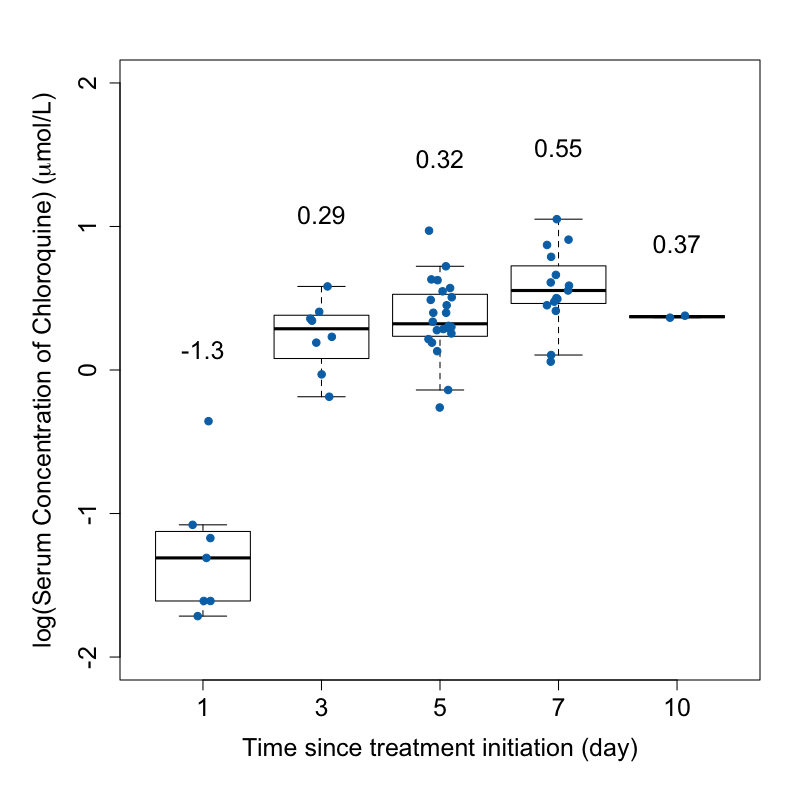

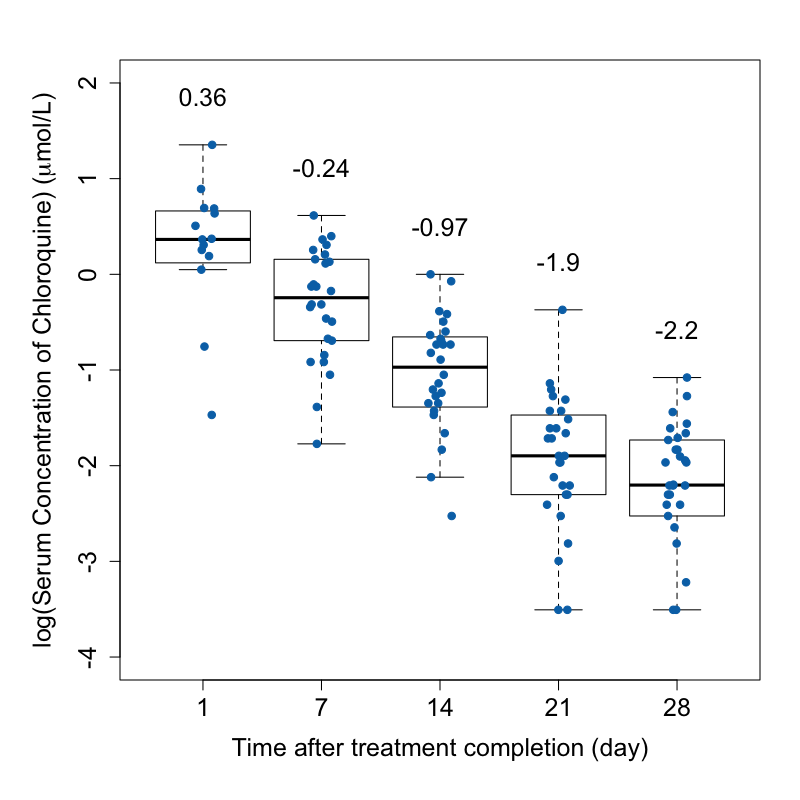
**

The text on top of each boxplot is representing the median value of log-transformed serum concentration of chloroquine.

**Supplementary Figure 4.** The Kaplan-Meier plot on the time to undetectable viral RNA stratified by tertiles of log-transformed maximum observed value of serum concentration of chloroquine (N=50; P=0.14).


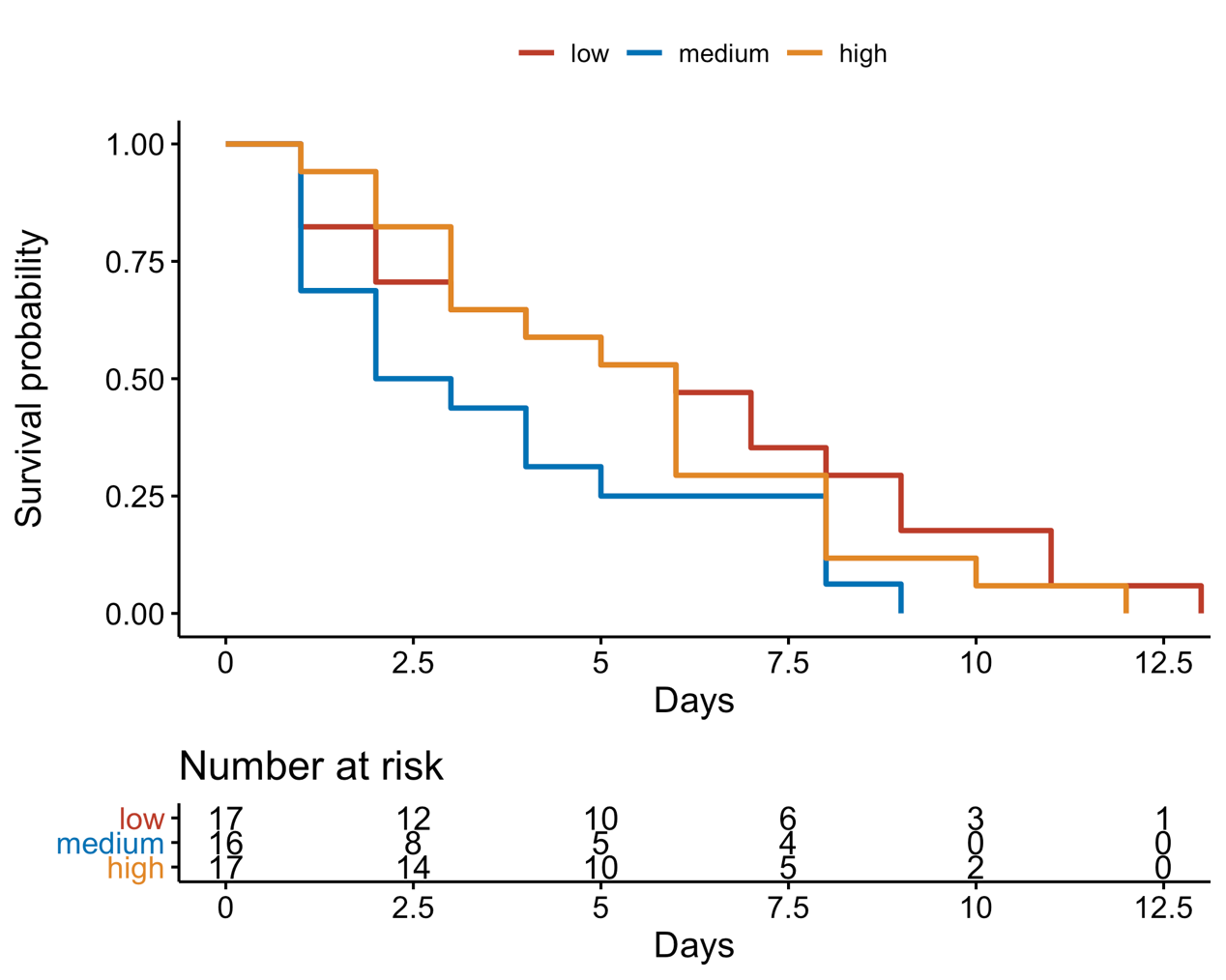


**Supplementary Methods**

**Definition of outcomes**

The negative conversion of viral RNA is defined as two consecutive SARS-CoV-2 RNA tests of respiratory tract specimens are both negative with an interval of at least 24 hours. The date of the negative conversion of viral RNA is defined as the first date of the two consecutive SARS-CoV-2 RNA tests. The primary outcome is defined as the day of initial treatment to the day of negative conversion of viral RNA. The secondary outcomes include the proportion of patients with undetectable viral RNA by day 10 and day 14, duration of fevers, the length of hospital stay. The duration of fevers is defined as the first day of fever substracting from the last day of fever with body temperature at least 37.3 ºC. The length of hospital stay is calculated by subtracting the day of hospital admission from the day of discharge.

**Measurement of serum concentration of Chloroquine**

High performance liquid chromatographic-tandem mass spectrometric (HPLC-MS/MS) is used to determine Chloroquine concentration in human serum. The blood samples were centrifuged and obtained serum were stored frozen (-20°C) until the serum concentration were determined. The chromatographic separation was on a Phenomenex Kinetex C18 (2.1 x 50 mm, 2.6 um) analytical column at 45°C. The mobile phase composition was consisted of A liquid (ddH_2_O, 10mM methanine and 0.5% formic acid) and B liquid (methanol, 10mM methanine and 0.5% formic acid), which was pumped at a flow rate of 0.4mL/min. The gradient elution of liquid chromatography was listed in **Supplemental Table 9**.

**Supplemental Table 9. The gradient elution of HPLC.**

| Time | flow rate (mL/min) | B liquid (%) |
| --- | --- | --- |
| 0.5 | 0.4 | 25 |
| 0.6 | 0.4 | 90 |
| 2.0 | 0.4 | 90 |
| 2.1 | 0.4 | 25 |
| 3.5 | 0.4 | 25 |

The tandem MS system (Waters Corporation) is equipped with an ESI source, and run with the Xcalibur 2.0 software (Waters Corporation). The mass spectrometer was operated in the positive ion and the source gas parameters were listed in the **Supplemental Table 10**. Multiple reaction monitoring (MRM) were listed in the **Supplemental Table 11**.

**Supplemental Table 10. Source gas parameter of MS.**

| Source gas parameter | Positive |
| --- | --- |
| Ion spay voltage | 5500 |
| Curtain gas | 35 |
| Nebulizer gas (gas 1) | 40 |
| Gas 2 | 40 |
| CAD | Medium |
| Interface heater temperature | 600 |

**Supplemental Table 11. The MS parameter of target molecular in positive ion model**.

| Q1 (Da) | Q3 (Da) | Time (msec) | ID | DP (Volt) | EP (Volt) | CE (Volt) | CXP (Volt) |
| --- | --- | --- | --- | --- | --- | --- | --- |
| 320.2 | 247.2 | 50 | CQ* | 90 | 10 | 50 | 5 |
| 320.2 | 142.2 | 50 | CQ | 90 | 10 | 45 | 5 |
| 292.2 | 179.2 | 50 | DCQ* | 90 | 10 | 30 | 5 |
| 292.2 | 114.2 | 50 | DCQ | 90 | 10 | 30 | 5 |
| 336.2 | 247.2 | 50 | HCQ | 90 | 10 | 57 | 5 |
| 336.2 | 158.2 | 50 | HCQ | 90 | 10 | 52 | 5 |

**Quantitative viral RNA testing**

We monitored SARS-CoV-2 RNA in nasopharyngeal swabs and feces among the inpatients with COVID-19 using the China CDC-standardized real-time reverse transcriptase polymerase chain reaction (rRT-PCR) from January 17, 2020 to March 3, 2020^1^. The copy numbers of SARS-CoV-2 RNA was indicated by the cycle threshold (CT) values of rRT-PCR with lower CT values corresponding to higher viral copy numbers. A CT value less than 40 is defined as positive as reported^2^.

**Original protocol and adverse events reporting guidelines**

1. This study is registered at Chinese Clinical Trial Registry (ChiCTR2000029609). The study protocol can be obtained from <http://www.chictr.org.cn/showproj.aspx?proj=49145>
2. Study flow
   1. Reason's for excluding patients on the 1st day of treatment.

According to the drug administration flow, the nasopharyngeal swabs for viral RNA testing is collected before medication administration in the morning. The result of viral RNA test will be obtained in the afternoon. Therefore, negative conversion of viral RNA in the patients on the first day of drug administration should not be considered due to the medication from the present study.

1. The following adverse events were monitored during chloroquine treatment in our study(1)
2. General adverse reactions: dizziness, headache, loss of appetite, nausea, vomiting, abdominal pain, acid reflux, belching, diarrhea, tinnitus, irritability, weight loss, fatigue, thirst, sleep disorder
3. Eye toxicity: (1) diffused white particles on cornea; (2) accumulated toxicity: mild retinal edema and pigment accumulation, blurred vision, and retinopathy (macular degeneration)
4. Severe external vertebral diseases: such as dystonia, dyskinesia, tongue extension, and torticollis
5. Cardiotoxicity: arrhythmia, chest tightness, pain under xiphoid, ventricular premature beat, shock, and Adams-Stokes syndrome (considered as severe adverse event).
6. Hematologic abnormalities: hemolytic anemia, aplastic anemia, agranulocytosis, thrombocytopenia
7. Others: drug-induced psychosis, depression, delirious, dysphoria, emotional unstable, leukopenia, purpura, rash, dermatitis, psoriasis, hair whitening, hair loss, neuromuscular pain, hand shanking/numberless, unsteady gait
8. an adverse event was identified, we would inform the research team through the adverse event reporting system, and evaluate whether to continue the chloroquine treatment based on the severity of the adverse event.

**References**

1. [Expert consensus on chloroquine phosphate for the treatment of novel coronavirus pneumonia]. *Zhonghua Jie He He Hu Xi Za Zhi*. 2020; **43**(0): E019.
